# Supplementary material for: Prevalence and Predictors of COVID-19 Vaccination Acceptance among Greek Health Care Workers and Administrative Officers of Primary Health Care Centers: A Nationwide Study Indicating Aspects for a Role Model
Source: Vaccines (Basel). 2022 May 12;10(5):765. doi: 10.3390/vaccines10050765 (PMC9142949; doi:10.3390/vaccines10050765)
Supplement: Supplementary file 1 [file vaccines-10-00765-s001.zip › vaccines-1684568-supplementary.pdf]

Supplementary material

S1 : Survey Questionnaire

UNIVERSITY OF THESSALY
SCHOOL OF HEALTH SCIENCES
FACULTY OF MEDICINE
DEPARTMENT OF HYGIENE & EPIDEMIOLOGY

QUESTIONNAIRE ON KNOWLEDGE, ATTITUDES AND PRACTICES OF HEALTH CARE PROFESSIONALS RELATED TO THE SARS-CoV-2 VACCINE

GENERAL SECTION

1. Age: …….. years
2. Gender: Male Female
3. Marital status (indicate with 'X'): Married Unmarried Other (please specify)……………

4. Education level (indicate with 'X'):
High school Institute of Vocational Training (IEK)
Technological Educational Institute (TEI) Higher Education Institute/University (AEI)
Master /Doctoral

5. Health care profession (indicate with 'X'):
Doctor (please indicate specialization ……………………………) Nurse
Medical Laboratory worker Other (please specify).............................

6. Sector of employment (indicate with 'X'):
Private hospital Public hospital
Health centre (K.Y.) Community-based primary health unit (To. M.Y)

7.Health District (Y.ΠE) of employment: ………………………………………………

8. Regional Unit of employment: …………………………………………..

9. Department of employment (indicate with 'X'): (please do not respond if you work at a health centre or community-based primary health unit)
Clinical Laboratory Other (please specify).............................

10. Section of employment (indicate with 'X'): (please do not respond if you work at a health centre or community-based primary health unit)
Pathology Surgery
Laboratory Other (please specify).............................

11. Years of practice: ....……………

PART A
12. Do you belong to a vulnerable/high risk group due to your medical history? (indicate with 'X'):
(cardiovascular disease, respiratory disease, diabetes, immunosuppression, cancer, pregnancy etc.)
YES NO

13. Do you live with older individuals or individuals belonging to a vulnerable/high risk group due to their medical history? (indicate with 'X'):
(cardiovascular disease, respiratory disease, diabetes, immunosuppression, cancer, pregnancy etc.)
YES NO

14. Please circle the choice that best indicates your response.

	Completely agree	Agree	Neither agree nor disagree	Disagree	Completely disagree
The HPV vaccine is recommended for all males up to 18 years of age in the country.	1	2	**3**	4	5
After the flu vaccination, certain foods are not permitted to be consumed for a period of 24 hours.	1	2	**3**	4	5
One of the contraindications of the flu vaccine is an allergy to eggs.	1	2	**3**	4	5

15. Please circle the choice that best indicates your response.

	Completely agree	Agree	Neither agree nor disagree	Disagree	Completely disagree
Vaccinations are an important tool for the protection of public health and in particular of health professionals and workers in the health sector	1	2	**3**	4	5
Natural immunity acquired via disease is always preferable to immunity acquired via vaccination.	1	2	**3**	4	5
Many vaccines often have serious side effects.	1	2	**3**	4	5

16. Are you the parent/guardian of one or more children? (indicate with 'X'):

YES NO

If **YES,** do you adhere to the child vaccination program suggested by the National Vaccination Program in the country? (please circle the answer of your choice)

YES, I vaccinate my children according to the National Vaccination Program

I select and carry out some vaccinations I do not vaccinate my children

17. Have you been vaccinated with the seasonal flu vaccine? (indicate with 'X'):

YES NO

If not, please indicate why (more than one response can be selected)

I do not have time
Apathetic
I use homeopathic remedies
I do not think I am at risk
Fear regarding vaccine safety
Other (please specify).............................

PART B

18. Do you know of a relative or friend who has had COVID-19? (indicate with 'X'):

YES NO

19. Do you come into contact with COVID-19 patients while performing your job duties? (indicate with 'X'):

YES NO

20. How do you evaluate your level of being informed about vaccines against the SARS-CoV-2 virus that causes COVID-19? (Please circle below the option which best represents your answer)

No information	Insufficient	Satisfactory	Excellent
1	2	3	4

21. Which channels do you use to keep informed about the COVID-19 pandemic and the SARS-CoV-2 vaccine, and how often? (please circle the option that represents your answer)

	Always	Often	Rarely	Never
Television	1	2	3	4
Social media channels (Facebook, Twitter, Instagram etc.)	1	2	3	4
Newspaper (in print or electronic versions)	1	2	3	4
General interest publications/journals (in print or electronic versions)	1	2	3	4
Medical articles in journals (in print or electronic versions)	1	2	3	4
Committee for infectious diseases at health facility	1	2	3	4
General interest websites	1	2	3	4
Website of the Hellenic National Public Health Organization (NPHO)	1	2	3	4
Website of the Hellenic Ministry of Health	1	2	3	4
Please specify other …………………………………………………………………..…..	1	2	3	4

22. Please circle the response below that represents your answer.

	Completely agree	Agree	Neither agree nor disagree	Disagree	Completely disagree
Some of the vaccines against SARS-CoV-2 which are approved and used in the country are based on mRNA technology	1	2	3	4	5
The dosage regimen of the vaccines against SARS-CoV-2 includes 3 doses	1	2	3	4	5
There is evidence that mRNA technology interferes with the DNA of cells	1	2	3	4	5

23. Have you been or will you be vaccinated with any of the vaccines against the SARS-CoV-2 virus which causes COVID-19, which have received the necessary approvals from the European Medicines Agency and the National Medicines Agency? (indicate with 'X'):

YES NO

If not, please indicate why (**indicate with 'X'**) (more than one response can be selected)

I do not have time

Apathetic

Fear regarding vaccine safety

I do not think I am at risk

I need further information in order to make a decision

Other (please specify) ……………………………………..………………………………………………

24. Does the short period of time for development of the vaccines cause you any concerns about its safety? (circle the option below that represents your answer)

Completely agree	Agree	Neither agree nor disagree	Disagree	Completely disagree
**1**	**2**	**3**	**4**	**5**

25. Do you believe that vaccination against SARS-CoV-2 should be mandatory for healthcare professionals? (indicate with 'X'):

YES NO

THANK YOU FOR YOUR TIME

S2 Table S6.

Table S6. Results of association between sources of information and correct knowledge / perceptions about vaccination and COVID-19 vaccines and Vaccination acceptance				
	**Proportional Ratio (PR) with 95% CI** (Always/Often vs Rarely/Never)			
	14a.The HPV vaccine is recommended for all males up to 18 years of age in the country, 14b. After the flu vaccination, certain foods are not permitted to be consumed for a period of 24 hours, 14c. One of the contraindications of the flu vaccine is an allergy to eggs **(all correct)**	15a Vaccinations are an important tool for the protection of public health and in particular of health professionals and workers in the health sector, 15b. Natural immunity acquired via disease is always preferable to immunity acquired via vaccination, 15c. Many vaccines often have serious side effects **(all Correct)**	22a. Some of the vaccines against SARS-CoV-2 which are approved and used in the country are based on mRNA technology, 22b. The dosage regimen of the vaccines against SARS-CoV-2 includes 3 doses, 22c. There is evidence that mRNA technology interferes with the DNA of cells **(all Correct)**	**Vaccination Acceptance**
Television	0.89 (0.82-0.96)	0.93 (0.82-1.06)	0.81 (0.73-0.90)	0.98 (0.93-1.03)
Social media	0.83 (0.77-0.90)	0.79 (0.70-0.91)	0.83 (0.75-0.93)	0.96 (0.91-1.01)
Newspaper (in print or electronic versions)	1.04 0.97-1.13)	0.99 (0.87-1.12)	1.12 (1.01-1.24)	1.05 (1.00-1.10)
General interest publications/journals	1.01 (0.93-1.11)	1.00 (0.86-1.15)	0.95 (0.84-1.07)	1.01 (0.95-1.06)
Medical articles in journals	1.43 (1.28-1.60)	2.00 (1.64-2.45)	1.78 (1.52-2.09)	1.15 (1.08-1.23)
Committee for infectious diseases at health facility	1.13 (1.05-1.22)	1.03 (0.91-1.17)	1.30 (1.18-1.44)	1.09 (1.04-1.15)
General interest websites	0.93 (0.85-1.01)	1.11 (0.99-1.23)	0.82 (0.73-0.92)	0.99 (0.94-1.04)
National Public Health Organization (NPHO)	1.33 (1.18-1.50)	1.61 (1.32-1.97)	1.89 (1.57-2.26)	1.19 (1.10-1.29)
Website of the Hellenic Ministry of Health	1.16 (1.06-1.26)	1.30 (1.13-1.50)	1.44 (1.28-1.62)	1.11 (1.05-1.18)

S3 Table S7.

Table S7. Results of association between demographical factors and other determinants of COVID-19 vaccine acceptance and correct knowledge and perceptions about vaccination and COVID-19 vaccines.				
		**Median (IQR) and p-value or Proportional Ratio (PR) with 95% CI**		
		14a.The HPV vaccine is recommended for all males up to 18 years of age in the country,<br>14b. After the flu vaccination, certain foods are not permitted to be consumed for a period of 24 hours,<br>14c. One of the contraindications of the flu vaccine is an allergy to eggs **(all correct)**	15a Vaccinations are an important tool for the protection of public health and in particular of health professionals and workers in the health sector,<br>15b. Natural immunity acquired via disease is always preferable to immunity acquired via vaccination,<br>15c. Many vaccines often have serious side effects **(all Correct)**	22a. Some of the vaccines against SARS-CoV-2 which are approved and used in the country are based on mRNA technology,<br>22b. The dosage regimen of the vaccines against SARS-CoV-2 includes 3 doses,<br>22c. There is evidence that mRNA technology interferes with the DNA of cells<br>**(all Correct)**
Age		Correct: 44 (12)<br>Incorrect:44 (15)<br>p=0.599	Correct: 44 (14)<br>Incorrect:44 (12.8)<br>p=0.209	Correct: 43 (13)<br>Incorrect:45 (14)<br>p=0.047
Gender	Male / Female	1.08 (1.00-1.17)	1.35 (1.19-1.53)	1.08 (0.97-1.20)
Marital status	Married	0.94 (0.86-1.02)	0.75 (0.66-0.86)	0.96 (0.86-1.07)
	Divorced	1.05 (0.86-1.29)	0.87 (0.60-1.27)	1.17 (0.92-1.49)
	Widowed	1.02 (0.58-1.80)	-	0.41 (0.08-2.25)
	NA	1.01 (0.84-1.22)	0.92 (0.68-1.26)	0.38 (0.22-0.66)
	Unmarried	Ref.	Ref.	Ref.
Educational level	Master /Doctoral	2.23 (1.56-3.21)	5.36 (2.31-25.80)	3.16 (1.97-5.07)
	Higher Education Institute/University (BSc, AEI)	2.25 (1.57-3.23)	6.35 (2.75-14.66)	2.5 (1.55-4.02)
	Technological Educational Institute (TEI)	1.57 (1.08-2.28)	3.13 (1.34-7.34)	1.79 (1.10-2.92)
	Institute of Vocational Training (IEK)	1.06 (0.68-1.66)	2.33 (0.92-5.85)	0.99 (0.54-1.80)
	High school	Ref.	Ref.	Ref.
Educational level (groups)	Higher Education Institute/University (AEI) & Master or Doctoral & Technological Educational Institute (TEI)	1.98 (1.60-2.46)	2.83 (1.96-4.09)	2.52 (1.87-3.38)
	High school& Institute of Vocational Training (IEK)	Ref.	Ref.	Ref.
Health care profession	Physician	1.48 (1.34-1.65)	2.52 (2.05-3.09)	1.68 (1.45-1.94)
	Health Consultant	1.23 (1.05-1.43)	1.83 (1.39-2.42)	1.55 (1.29-1.88)
	Other Health Professionals	0.84 (0.67-1.05)	1.16 (0.82-1.66)	1.12 (0.88-1.43)
	Administrative	0.99 (0.82-1.21)	0.82 (0.54-1.26)	1.00 (0.77-1.31)
	Nursing staff	Ref.	Ref.	Ref.
Health care profession (groups)	Physician+ Health Consultant	1.49 (1.37-1.63)	2.40 (2.04-2.82)	1.61 (1.44-1.81)
	Nursing staff+ Other Health Professionals + Administrative	Ref.	Ref.	Ref.
Sector of employment	Health Center	0.92 (0.83-1.02)	0.98 (0.82-1.18)	0.77 (0.69-0.86)
	Local Health Unit	Ref.	Ref.	Ref.

Health District of employment (Υ.ΠΕ)	1st	1.12 (0.94-1.33)	0.88 (0.63-1.22)	1.57 (1.29-1.92)
	2nd	1.09 (0.92-1.29)	0.90 (0.67-1.22)	1.48 (1.21-1.81)
	3rd	1.07 (0.94-1.23)	0.82 (0.64-1.04)	1.27 (1.06-1.53)
	5th	0.92 (0.80-1.06)	0.87 (0.70-1.08)	1.24 (1.04-1.48)
	6th	1.23 (1.09-1.38)	1.37 (1.14-1.63)	1.09 (0.90-1.31)
	7th	2.11 (1.10-4.05)	0.95 (0.70-1.30)	1.50 (1.22-1.92)
	4th	Ref.	Ref.	Ref.
Health District of employment (Υ.ΠΕ) (groups)	3,4,5	0.83 (0.77-0.90)	0.77 (0.68-0.87)	0.92 (0.82-1.02)
	1,2,6,7	Ref.	Ref.	Ref.
Years of practice		Correct:14 (17)<br>Incorrect:14 (18)<br>p=0.766	Correct:13 (16)<br>Incorrect:15 (18)<br>p=0.215	Correct:14 (16)<br>Incorrect:14 (18)<br>p=0.838
Q17 Have you been vaccinated with the seasonal flu vaccine?	Yes/No	1.26 (1.16-1.37)	1.31 (1.23-1.39)	1.24 (1.16-1.33)
Q24. Does the short period of time for development of the vaccines cause you any concerns about its safety?	Disagree/Agree	1.3 (1.17-1.44)	1.45 (1.34-1.57)	1.37 (1.25-1.49)
Q25. Do you believe that vaccination against SARS-CoV-2 should be mandatory for healthcare professionals?	Yes/No	1.3 (1.17-1.44)	1.37 (1.23-1.53)	1.34 (1.19-1.51)
